# Exosomal microRNAs as Early Transition Biomarkers from Recurrent-Remissive to Secondary Progressive Multiple Sclerosis

**DOI:** 10.3390/ijms26083889

**Published:** 2025-04-20

**Authors:** Oana Mosora, Smaranda Maier, Doina Manu, Laura Bărcuțean, Medeea Roman, Mihai Dumitreasă, Rodica Bălașa

**Affiliations:** 1 Doctoral School, “George Emil Palade” University of Medicine, Pharmacy, Science, and Technology of Targu Mures, 540142 Targu Mures, Romania; oanamosora.92@yahoo.com (O.M.); rodica.balasa@umfst.ro (R.B.); 2 Ist Neurology Clinical, Emergency Clinical County Hospital Targu Mures, 540136 Targu Mures, Romania; laurabarcutean@gmail.com (L.B.); r.medeea@yahoo.com (M.R.); mihai.du96@gmail.com (M.D.); 3 Department of Neurology, “George Emil Palade” University of Medicine, Pharmacy, Science, and Technology of Targu Mures, 540136 Targu Mures, Romania; 4 Center for Advanced Medical and Pharmaceutical Research, “George Emil Palade” University of Medicine, Pharmacy, Science, and Technology of Targu Mures, 540142 Targu Mures, Romania; doina.manu@umfst.ro

**Keywords:** multiple sclerosis, miRNAs, exosomes, relapsing-remitting multiple sclerosis, secondary progressive multiple sclerosis, biomarker

## Abstract

Multiple sclerosis (MS) is a chronic, immune-mediated disease that affects young adults, leading to neurological disability. Regardless of the studies and the research involved in developing an efficient disease-modifying therapy (DMT), relapsing-remitting multiple sclerosis (RRMS) will transition to a progressive multiple sclerosis phenotype. The moment of transition from RRMS to secondary progressive multiple sclerosis (SPMS) is difficult to predict, and the diagnosis is based on the accumulation of disabilities in the evolution of the disease. Research on microRNAs’ (miRNAs) role in MS began in the early 2000s, with miR-155 frequently cited for its link to blood–brain barrier dysfunction and neurodegeneration, making it an early transition biomarker from RRMS to SPMS. The purpose of this review is to reveal the importance of finding a biomarker from the molecular field that will be able to identify the transition phase so patients can receive high-efficacy treatments and to cease the clinical progression.

## 1. Introduction

Multiple sclerosis is a chronic, inflammatory, and neurodegenerative disease that affects the central nervous system (CNS), and is one of the most important conditions that lead to neurological disability in young adults [1]. A total of 2.8 million people worldwide (35.9 per 100,000 population) are suffering from MS, according to the latest data [2]. Complex genetic, epigenetic, microbial, and environmental factors are involved in MS physiopathology [3]. The autoimmune processes involved in this disorder cause progressive loss of neurological function due to the damage of the axonal myelin sheath in the brain and the spinal cord [4]. The persistent inflammatory state is maintained by immune cells, such as CD8+ T cells, CD4+ T cells, and B cells. Moreover, during the disease course, the blood–brain barrier (BBB) is disrupted, which allows Th1 and Th17 to cross into the CNS, creating a pro-inflammatory environment by secretion of cytokines, such tumor as necrosis factor α (TNF α), IL-1, IL-6, and interferon-γ (IFN-γ) [5].

Clinically, we can differentiate several subtypes of MS. RRMS is the most common phenotype, affecting 85–90% of patients, while primary-progressive multiple sclerosis (PPMS) occurs in 10–15% of patients [6]. In RRMS, patients will have exacerbations lasting from days to weeks, with subsiding of symptoms, full or partial recovery, and no disease progression between attacks [7]. In time, RRMS will progress to SPMS, defined by accumulation of disability with or without relapses. PPMS is characterized by continue accumulation of neurological disability, without relapses [8]. The identification of magnetic resonance imaging (MRI) lesions in asymptomatic patients is defined as radiologically isolated syndrome [9]. In modern research, new terms have been described that have neurodegeneration as the key player for progressive forms of MS [10]. “Progression independent of relapse activity” (PIRA) was first described by Kappos and colleagues [11]. They mentioned a disability progression in patients with RRMS appearing in a period free of relapses, which is not influenced by a remaining disability from a previous relapse [12]. “No evidence of disease activity” (NEDA) is described as no disease activity (no relapses or new enhancing lesions on the MRI), but the criteria that need to be considered in this stage are loss of brain volume, cerebrospinal fluid (CSF) light chain neurofilaments, or cognitive decline [13]. The progressive clinical evolution in patients with no MRI Gadolinium positive activity is considered secondary to smoldering demyelination (“silent progression”) in connection with brain atrophy [14,15].

The pathological processes are different between relapses and progression stages of MS [16]. If the initial mechanism for RRMS patients is the inflammation process and BBB damage, neurodegeneration and brain atrophy play the key roles for SPMS patients at this stage of the disease, with these factors being responsible for the irreversible progression of the disability [17,18].

The diagnosis of MS is based on a combination of the patient’s medical history, presenting symptoms, physical examination, and a range of paraclinical tests. These include CSF analysis for the presence of oligoclonal bands, MRI to detect inflammatory lesions, and blood tests. Together, these assessments form the basis of the 2017 McDonald criteria for diagnosing MS [19]. However, the moment of transition from RRMS to SPMS is difficult to diagnose, and the diagnosis is based on the accumulation of disabilities in the evolution of the disease [20]. The most important criteria for the diagnosis of SPMS is the increase by at least one point in the Expanded Disability Status Scale (EDSS) for patients that initially had an EDSS score ≤ 5.5 points, or 0.5 points if the initial score was ≥ 6.0 points [21].

It is important for clinicians to be able to differentiate the clinical phenotypes of MS. To be able to use a personalized treatment in MS, it will be helpful to have biomarkers that are able to distinguish the level of progression of the disease and the response to treatment [22,23].

## 2. miRNAs—Biological Origins

miRNAs are characterized by stability, easy extraction from body fluids, and the ability to change the expression profile in the early stages of the disease, making them perfect tools to potentially diagnose and create prognostic factors [24]. Endogenous non-coding RNA (ncRNA) comprises RNA molecules transcribed within cells that do not encode proteins but serve crucial regulatory functions in gene expression, chromatin remodeling, RNA processing, and cellular activities. Based on their length, they are classified into two main categories: miRNAs—short, single-stranded ncRNAs (21–25 nucleotides) that regulate mRNA expression at the post-transcriptional level; and long non-coding RNAs (lncRNAs) (>200 nucleotides), which influence gene expression at both transcriptional and post-transcriptional levels by interacting with DNA, RNA, or proteins, playing roles in chromatin remodeling, mRNA stability, and cellular signaling pathways [25].

The first form primary miRNA (pri-miRNA) interacts with RNA polymerase II to form a stem–loop structure (pre-miRNA) under the action of ribonuclease III Drosha in the nucleus. This is transported with the aid of exportin-5 to the cytoplasm [26]. Pre-miRNAs are converted by ribonuclease III Dicer into mature miRNAs. Mature miRNAs in association with argonaute protein will form the miRNA-associated silencing complex that silences target genes through repressing mRNA translation or degrading the mRNA [27,28]. The miRNA regulatory mechanism is involved in biological processes such as differentiation, proliferation, inflammation, and cell apoptosis [29]. Also, they are involved in myelin repair, neurogenesis, and gliogenesis. Dysregulation of miRNAs is clearly involved in autoimmune processes and neurodegeneration [30,31].

miRNAs are present in biological fluids like saliva, blood, plasma, serum, and CSF, where they are protected from RNase degradation by associating with proteins or being enclosed in extracellular vesicles such as exosomes. However, studies indicate that 83% to over 99% of miRNAs can be found within the exosomal fraction, highlighting their predominant vesicular transport [32]. Moreover, exosomes are one of the most important biomarkers of the future because of their easy extraction from body fluids [33]. Being enclosed in the membrane vesicles makes the miRNAs extremely stable, even with high RNase activity [34]. Different studies demonstrated strong resistance to stressful conditions, such as prolonged storage time, temperature difference, and repeated freeze–thaw cycles, making miRNAs good diagnostic and prognostic biomarkers [35,36].

## 3. Early miRNA Biomarkers Explored in MS

Researchers have shown the important role that miRNAs have in neuronal development, synaptic plasticity, nervous system morphogenesis, and neurodegeneration [37]. All these processes are affected during the MS course. The very first research on miRNA’s importance in the development of MS started back in the early 2000 [38]. The dysregulation of miR-142-3p, miR-146a, miR-145, miR-155, miR-22, miR-223/3p, and miR-326 has been consistently reported in the literature, suggesting a shared role in the modulation of immune pathways relevant to autoimmune diseases such as MS [39]. The most constantly mentioned miRNA in the studies is miR-155, which was associated with dysregulation of the blood–brain barrier and neurodegeneration [40].

Another important miRNA that was found with regularity to be dysregulated in MS patients is miR-21, which was found to be up-regulated during active disease and down-regulated in remission [41]. Furthermore, the let-7 family was found to be dysregulated in the immune compartment and miR-103 was down-regulated in all cellular compartments [42,43].

To obtain more insight into the specific cellular miRNA involved in MS, we performed an analysis of the studies that mention the differentially expressed miRNAs and their implication in MS physiopathology [44].

## 4. Dysregulated miRNAs Implicated in MS Pathology

In several studies it was shown that activity of miRNAs in the development of immune system cells and in the differentiation and activation of B and T lymphocytes is essential, as illustrated in Table 1. T cell subsets, like Th1 and Th17, are important mediators of inflammation during the cellular immune response [45]. O’Connell et al. showed that miR-155 inhibits suppressor of cytokine signaling-1 (SOCS1) in activated CD4^+^ T cells and promotes the differentiation of Th1/Th17 cells [46].

MiR-17-5p, a microRNA associated with autoimmunity, was found to be up-regulated in CD4(+) cells from individuals with MS, as reported by Lindberg et al. This up-regulation was linked to changes in the expression of its predicted target genes—phosphatase and tensin homolog (PTEN) and phosphatidylinositol-3-kinase regulatory subunit 1 (PIK3R1)—both of which were down-regulated following in vitro stimulation of CD4(+) cells with anti-CD3/CD28. Functional studies using a synthetic miR-17 inhibitor further supported the connection between miR-17 expression and the suppression of these target genes. Additionally, stimulation elicited distinct responses from deregulated miRNAs—miR-17-5p and miR-193a—showed marked up-regulation, while miR-497, miR-1, and miR-126 were down-regulated [47].

Differentiation of Th1 and Th17 was reduced by silencing let-7e. In mice with experimental autoimmune encephalomyelitis (EAE), it was shown that let-7e was up-regulated in CD4^+^ T cells and CNS inflammatory infiltrating monocytes and also regulated the expression of IL-10 and IL-13. Guan et al. concluded that let-7e polarized CD4^+^ T cells into Th1 and Th17 cells, participating in the pathogenesis of EAE [48]. Overexpression of miR-30a had shown the inhibition of Th17 differentiation. Moreover, it was found that patients with MS have low levels of miR-30a in CD4^+^ T cells [49].

miR-146 was shown to reduce and inhibit the adhesion of T cells to cerebral vascular endothelial cells, having an important role in the development of MS and EAE [50]. In MS patients, up-regulation of miR-34a led to elevating Th17 cells and suppressed the formation of Treg cells and differentiation toward Th17 cells [51]. Nakahama et al. found that increased expression of miR-132 promotes the differentiation of Th17 cells, leading to progression of EAE [52].

**Figure 1:**
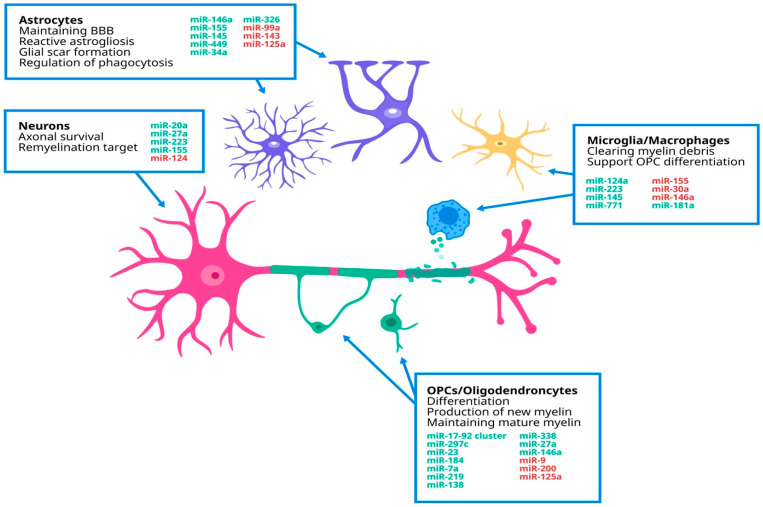
Dysregulated miRNAs in correlation with the key cells in the CNS. We illustrate the key cells in MS pathophysiology: neurons, whose axonal destruction drives clinical outcomes; astrocytes, which support the blood–brain barrier (BBB) and contribute to scar formation; macrophages, which clear debris; and oligodendrocyte progenitor cells (OPCs), which differentiate to sustain mature myelin. The up-regulated miRNAs are listed in green and the down-regulated miRNAs are listed in red.

Neurons are one of the most important elements in MS’s evolution because of the demyelinating axons that are not able to transmit the electrical signal, with the process ending in cell death (Figure 1) [53]. Studies showed that miRNAs are important in the survival of the axon. Morquette et al. affirmed that miR223 and miR-27a are up-regulated following local damage and are associated with neuroprotection from glutamate toxicity, and are also involved in microglial remyelination [54].

In brain tissue samples, isolated miR-124 was detected in demyelinating hippocampal neurons. Its reduced expression correlated with memory dysfunction and was linked to decreased levels of ionotropic receptors AMPA 2 and AMPA 3, which mediate most excitatory synaptic transmission in the central nervous system and play a crucial role in synaptic plasticity, which is essential for learning and memory [55].

In the EAE model, the down-regulation of miR-125a, combined with the activation of vitamin D receptors, was associated with improved clinical outcomes. Additionally, studies in mice showed that the up-regulation of miR-155 and miR-20a is crucial for neuronal survival, suggesting a potential neuroprotective role [56,57].

Astrocytes are the most numerous glial cells in the CNS, having important functions: maintaining the function of the BBB, providing energy and metabolic support to neurons, and supporting synaptic transmission [58]. Rao et al. found that in MS lesions, miR-145, miR-99a, and miR-143 are down-regulated in astrocytes in active grey lesions and white matter lesions, while miR-449 and miR-125a are up-regulated in white matter lesions. All these miRNAs are involved in astrogliosis, being modulators of glial scar formation [59]. MiR-34a and miR-326 are involved in microglia/macrophage phagocytosis, determining the clearance of myelin debris [60]. Finally, miR-146a was shown to be down-regulated in active lesions, thus suggesting that astrocytes are not involved into lesion formation [61].

The innate immune system of the CNS is represented by microglia that are macrophage-like cells. Their role is to respond to infection or damage that could occur in the brain or the spinal cord [61]. Martin et al. pointed out that miR-146a is associated with reduced microglia numbers, but also increases anti-inflammatory activation of the microglia [62]. miR-145 and miR-771 are closely linked to the anti-inflammatory activation of microglia, while the down-regulation of miR-155 appears to suppress inflammatory cytokine production. miR-155 plays a key role in microglial activation, promoting the release of pro-inflammatory factors such as TNF-α, IL-1β, IL-6, interferon-inducible protein 10 (IP-10), macrophage inflammatory protein-1α (MIP-1α), monocyte chemoattractant protein-1 (MCP-1), and nitric oxide (NO) [63]. Up-regulation of miR-30a is involved in the secretion of factors that promote apoptosis for the oligodendrocyte progenitor cells (OPCs) [64].

In the CNS, the myelin is produced by the oligodendrocytes, but in MS it is important in the recruitment of OPCs that are capable of differentiating into mature, myelinating oligodendrocytes [65,66]. Dugas et al. showed that miR-219, miR-138, and miR-338 are up-regulated during OPC differentiation, supporting remyelination. miR-219 is implicated in both stages of OPC differentiation, while miR-138 promotes early differentiation while delaying later stages. All these miRNAs have been found in white matter lesions of PPMS [58]. Up-regulation of miR-9 and miR-200 affects OPC differentiation [67]. Another important miRNA is miR-23, which provides full maturation of the oligodendrocytes and has a role in the myelin maintenance [68]. Up-regulation of miR-7a promotes generation of oligodendrocyte lineage cells, while stuttering maturation [69]. Several miRNAs are actively implicated in the OPC differentiation and remyelination, such as miR-146a, miR-17-92 cluster, and miR-297c [70,71,72].

**Table 1:** miRNAs correlated with the cells involved in MS pathogenesis.

Cell Type	miRNA	Role
**CD4^+^T cells**	miR-34a	-⇓ related to the late stage of neuron mitosis [51]
	miR-146a	-⇓ regulates T cell adhesion [50]
	Let-7e	- inhibits the proliferation of Th2 cells and makes polarization of CD4+ T cells towards Th1 cells and Th17 cells [48]
**Th 1 and Th 17 cells**	miR-155	-⇑ promotes the down-regulation of CD47 expression, and triggers macrophage-mediated myelin phagocytosis [56]
	miR-30a	- negatively regulates the expression level of IL-17 [49]
	miR-132	- promotes the differentiation of Th17 cells [52]
**CD ^8+^ B cells**	miR-17-92	- affects phosphatidylinositol-3 kinase in B-cell receptor [57]
	miR-106b	
**Neuron**	miR-155	-neuroprotective [56]
	miR-20a	
	miR-124	-⇑ regulation demyelination of axons [55]
	miR-223	-⇑ regulation neuroprotective [54]
**Astrocyte**	miR-146a	-reduces astrocyte activation [59]
	miR-155	-expression in astrocytes releases microglia of inhibitory control of phagocytosis [56]
	miR-145	-inhibits astrogliosis [59]
	miR-99a	-⇓ regulation inhibits astrocyte proliferation [59]
	miR-143	-⇓ regulation inhibits astrocyte proliferation [59]
	miR-449	-attenuates glial scar formation [59]
	miR-34a	-expression in astrocytes releases microglia of inhibitory control of phagocytosis [60]
	miR-326	
**Microglia**	miR-124	-promotes inactive state [55]
	miR-223	-phagocytosis of myelin debris [54]
	miR-30a	-⇑ regulation promotes release of factors that induce OPC apoptosis [64]
	miR-145	-associated with anti-inflammatory microglia activation [63]
	miR-771	
	miR-146a	-promotes anti-inflammatory activation [62]
**Oligodendrocyte**	miR-219	-maintain mature myelin sheath [58]
	miR-138	-promotes OPC differentiation [66]
	miR-338	
	miR-125a	
	miR-27a	-proliferation and differentiation of OPC [66]
	miR-146a	-promotes OPC differentiation [70]
	miR-17-92-cluster	
	miR-297c	
	miR-9	-⇑ regulation impairs differentiation [67]
	miR-200	
	miR-23	-supports myelin maintenance [68]
	miR-7a	-promotes generation of OPCs [69]

## 5. Potential miRNAs That Could Differentiate RRMS from SPMS

In recent years, clinical studies (Table 2) proved that the dysregulation of miRNAs can be used as an early biomarker of conversion from RRMS to SPMS.

Exosomes represent a way of communication between the peripheral immune system and the CNS cells. In MS, the exosomes are one of the methods of interplay between the central and peripheral compartments [73,74]. The study conducted by Ebrahimkhani et al. evaluated miRNAs detected from exosomes. They included in the study 14 RRMS patients, 7 SPMS patients, 4 PPMS patients, and 11 healthy individuals. They identified differentially expressed exosomal miRNAs in RRMS (miR-15b-5p, miR-451a, miR-30b-5p, and miR-342-3p) and in progressive MS patients (miR-127-3p, miR-370-3p, miR-409-3p, and miR-432-5p) in relation to the control group. Moreover, they profiled a group of nine miRNAs that distinguished relapsing-remitting from progressive disease (miR-15b-5p, miR-23a-3p, miR-223-3p, miR-374a-5p, miR-30b-5p, miR-433-3p, miR-485-3p, miR-342-3p, and miR-432-5p). The result of the study showed that exosomal miRNAs are biomarkers that can be used as a diagnostic tool, but also could predict the disease subtype [75].

**Table 2:** Studies regarding miRNAs’ role as biomarkers differentiating RRMS from SPMS.

Study	Reported Study Population	Biological Material	Selected Reported Results
Ebrahimkhani et al., 2017 [75]	14 RRMS, 7 SPMS, 4 PPMS, 11 health controls (HCs)	Exosomes	-Nine miRNAs were identified with a different expression between RRMS and progressive MS patients: miR-15b-5p, miR-23a-3p, miR-223-3p, miR-374a-5p, miR-30b-5p, miR-433-3p, miR-485-3p, miR-342-3p, miR-432-5p). -A combination of miR-223-3p, miR-485-3p and miR-30b-5p showed a 95% accuracy rate in distinguishing progressive forms of MS from RRMS.
Niwald et al., 2017 [76]	13 RRMS-relapse, 23 RRMS- no relapse in 2 years	Exosomes	-Decreased expression of miR-155 and miR-301a (in 94% and 51% of samples, respectively) and an increased expression of miR-326 (in 72% samples) in RRMS patients. -Levels of miR-301a and miR155 were higher in RRMS patients in post-acute vs. stable phase of remission.
Selmaj et al., 2017 [77]	19 RRMS, 10 HCs	Exosomes	-miR-122-5p, miR-196b-5p, miR-301a-3p, and miR-532-5p decreased during relapse.
Kacperska M., 2015 [78]	37 RRMS, 20 HC	Exosomes	-Expression levels of miR-let-7a in patients in remission were lower than in the control group. - miR-648a in patients in MS remission was lower than in the control group.
Kramer et al., 2019 [79]	218 MS patients, 211 patients with other neurological diseases (OND)	Cerebral spinal fluid	-Up-regulation of miR-181 in patients with MS vs. patients with OND; no difference in patients with RRMS and SPMS, but difference between SPMS and PPMS. -Up-regulation of miR-633 in patients with MS vs. OND; higher levels in SPMS than in PPMS.
Haghikia et al., 2012 [80]	53 MS patients (17 RRMS, 30 SPMS, 6 PPMS), 39 OND	Cerebral spinal fluid	-miR-922 (*p* = 0.0001) was down-regulated, whereas miR-181c (*p* = 0.0007) and miR-633 (*p* = 0.0014) were up-regulated as compared with OND.
Ibrahim et al., 2020 [81]	39 RRMS, 35 SPMS, 10 HC	Serum	-miR-300 and miR-450b-5p expressions were significantly lower in SPMS patients, regardless of the EDSS score, compared with RRMS patients with EDSS 5.0.
Sharaf-Eldin et al., 2017 [82]	19 RRMS, 18 SPMS, 10 NMSOD, 23 HC	Serum	-miR-145 and miR-223 up-regulated in MS patients compared to healthy subjects. -miR-326 showed insignificant up-regulation, but is significantly lower in SPMS, compared with RRMS with EDSS < 5.0.
Gandhi et al., 2013 [83]	10 RRMS, 9 SPMS, 9 HC	Serum	-miR-92a-1, miR-135a, miR-454, miR-500, and miR-574-3p showed a significant association with RRMS. -let-7d, miR-145, and let-7c showed an association with SPMS.
Geiger et al., 2023 [84]	29 RRMS, 14 SPMS, 7 PPMS	Serum	-miR-143-3p levels were significantly lower in the SPMS. -higher miR-92a-3p and miR-486-5p levels were associated with greater total white matter lesion volumes within the cervical spine.
Vistbakka J., 2016 [85]	31 PPMS, 31 SPMS, 21 HC	Serum	-miR-191-5p showed the strongest up-regulation in progressive MS. -up-regulation of miR-128-3p and miR-24-3p in PPMS. -up-regulation of miR-375 in SPMS.
Regev et al., 2016 [86]	Discovery phase: 7 RRMS, 9 SPMS, 10 PPMS, 20 HC Validation phase: 29 RRMS, 19 SPMS, 10 PPMS, 30 HC	Serum	-miR-27a-3p and miR-376b-3p were the only miRNAs showing significantly different expressions between RRMS and SPMS. After multiple comparisons only miR-27a-3p remained significant.
Regev et al., 2018 [87]	Discovery phase: 7 RRMS, 9 SPMS, 10 PPMS, 20 HC Validation phase: 29 RRMS, 19 SPMS, 10 PPMS, 30 HC Reproductibility phase: 24 RRMS, 18 SPMS, 30 HC Transportability phase: 91 RRMS, 33 SPMS, 58 HC	Serum	-Significant down-regulation of miR-337-3p in patients with SSPMS compared with RRMS
Agostini et al., 2023 [88]	33 RRMS, 36 RRMS patients converted in SPMS patients, 30HCs -10-year observation period	Serum	-miR34a-5p, miR-103a-3p, and miR-376a-3p are significantly more expressed in RRMS that will convert to SPMS within 10 years compared to RRMS.
Al-Temaimi et al., 2024 [89]	76 MS, 75 HCs	Serum	-miR-24-3p was down-regulated in all MS patients compared to healthy controls. -miR-484 was significantly up-regulated in RRMS patients compared to HCs. -mir-146-5p and miR-484 were significantly down-regulated in SPMS compared to RRMS.
Gonzalez-Martinez et al., 2023 [90]	144 MS patients: 104 benign, 40 not benign with a 10- year follow-up	Serum	-Patients who retained benign MS had lower values of miR-25-3p (*p* = 0.047) and higher miR-320b (*p* = 0.025) values. -Development of SPMS was associated with higher miR-320b (*p* = 0.002) levels. -Brain parenchymal fraction at year 10 was negatively correlated with miR-25-3p (*p* = 0.0004) and positively correlated with miR-320b (*p* = 0.006).
Mohammadinasr et al., 2023 [91]	30 RRMS untreated patients, 30HCs	Cerebral spinal fluid and serum exosomes	-Let-7 g-5p, miR-18a-5p, miR-145-5p, miR-374a-5p, miR-150-5p, and miR-342-3p were significantly up-regulated in both CSF and serum-derived exosomes of RRMS patients compared to corresponding HCs. -miR-132-5p and miR-320a-5p were significantly down-regulated in both CSF and serum-derived exosomes of RRMS patients compared to HCs.

Niwald et al. aimed to estimate the levels of miR-155, miR-326, and miR-301 isolated from serum exosomes of patients with RRMS at different phases of the disease. They included 36 patients with RRMS at different phases of the disease (immediately after a relapse, and patients with no relapses in over 2 years). They observed that expression levels of miR-301a and miR-155 were higher in patients in the post-acute phase versus the stable phase of remission. Additionally, miR-155 was decreased in patients with EDSS > 5.0 and miR-326 was increased in patients with EDSS ≤ 5 [76].

The study of Selmaj et al. showed the profiling of exosomal miRNAs in patients with RRMS. The research included 101 MS patients and 51 HCs, and this analysis yielded four miRNAs that were dysregulated for patients with RRMS compared to HCs (miR-122-5p, miR-196b-5p, miR-301a-3p, and miR-532-5) [77].

There is also Kacperska et al.’s study, which included 20 patients undergoing relapse, 17 patients in remission, and 30 HCs. miR-let-7a had a statistically significant difference in expression across the HCs, and had a lower expression in the group of patients in remission. A low level of miR-92a expression tended to correlate in the group of patients in remission, while miR-648a expression was higher in the relapse group of patients [78].

To validate a previously discussed miRNA panel in the CSF, Kramer et al. analyzed 218 patients with MS and 211 with other neurological diseases (ONDs). It was demonstrated that miR-181-c was dysregulated and different between RRMS and SPMS. CSF levels of miR-633 were higher in patients with SPMS than PPMS, but did not differ between RRMS and SPMS patients [79]. Another study that also examined the patient’s CSF was the one conducted by Haghikia et al. They included in the study 53 MS patients (17 RRMS, 30 SPMS, and 6 PPMS) and 39 patients with ONDs. Analysis of miRNAS showed low levels of miR-633 and miR-181c in SPMS compared with RRMS [80].

Ibrahim et al. examined miR-450b-5p, miR-300, and Rho-associated protein kinase (ROCK2) as potential biomarkers to discriminate between RRMS and SPMS. The study included 39 RRMS cases, 35 SPMS cases, and 10 HCs. The patients were subdivided into two subgroups: EDSS ≤ 5.0 or EDSS > 5.0. The ROCK2 level in SPMS patients was found to be higher than that of RRMS patients. Additionally, miR-300 and miR-450b-5p levels were lower in SPMS compared with RRMS [81].

Another study explored the idea of biomarkers that would be used for differential diagnosis in immune-mediated neuroinflammatory diseases, such as neuromyelitis optica spectrum disorder (NMOSD), neuropsychiatric systemic lupus erythematosus (NPSLE), or MS. A total of 37 MS patients were analyzed (18 RRMS and 19 SPMS), 10 NMOSD patients, 10 NPSLE patients, and 23 HCs in a study conducted by Sharaf-Eldin et al. The use of the combinations miR-145/miR-326, miR-223/miR-326, and miR-145/miR-223/miR-326 could differentiate between RRMS and SPMS [82].

In another study, Gandhi et al. explored the miRNAs from the let-7 family that are expressed in MS. In the study they analyzed 368 miRNAs from the plasma of 10 RRMS patients, 9 SPMS patients, and 9 HCs. The expression of miR-92a-1-3p, let-7c, let-7d, and miR-145-5p was significantly up-regulated in the plasma of RRMS patients in comparison to SPMS patients (*p* = 0.022, *p* = 0.04, *p* = 0.047, *p* = 0.01). Moreover, miR-92a-1-3p showed a significant association with RRMS, whereas SPMS had no links. The let-7 family is involved in stem cell differentiation and T cell activation and is linked to neurodegeneration [83].

The dysregulation of miRNAs was also shown to be correlated with MRI measurements. Geiger et al. examined 29 RRMS, 14 SPMS, and 7 PPMS and showed a significant association between mir-92a-3p and miR-486-5p and lesion volumes within the cervical spine (higher expression of miRNAs, meaning higher lesion volume). The study also showed that miR-143-3p levels were significantly lower in the SPMS [84].

More and more studies concentrate on the differentiation between RRMS and progressive MS. In 2016, Vistbakka et al. studied the dysregulation of miRNAs in a study that included 31 SPMS patients, 31 PPMS patients, and 21 HCs. They showed that miR-191-5p was overexpressed in both progressive subtypes of MS, while miR-376c-3p, miR-128-3p, and miR-24-3p were up-regulated only in PPMS. Progression index correlated miR-375 with SPMS, and this finding makes it possible to assess the neurological progression [85].

In another study, Regev et al. validated seven miRNAs that can be used as biomarkers for the diagnosis of MS and two other miRNAs that are linked to disease progression. This study had a discovery phase that included 7 RRMS patients, 9 SPMS patients, 10 PPMS patients, and 20 HCs, and a validation phase with 19 SPMS, 10 PPMS, 29 RRMS, and 30 HCs. miR-27a-3p and miR-376b-3p were the only miRNAs showing significantly different expressions between RRMS and SPMS. miR-199a-5p was correlated with the EDSS, having the strongest association with disability [86]. The same authors found that miR-337-3p in serum is significantly down-regulated in SPMS compared to RRMS. Thus, we can consider that miR-337-3p has the potential to be a biomarker for disability and disease progression in MS patients [87].

**Figure 2:**
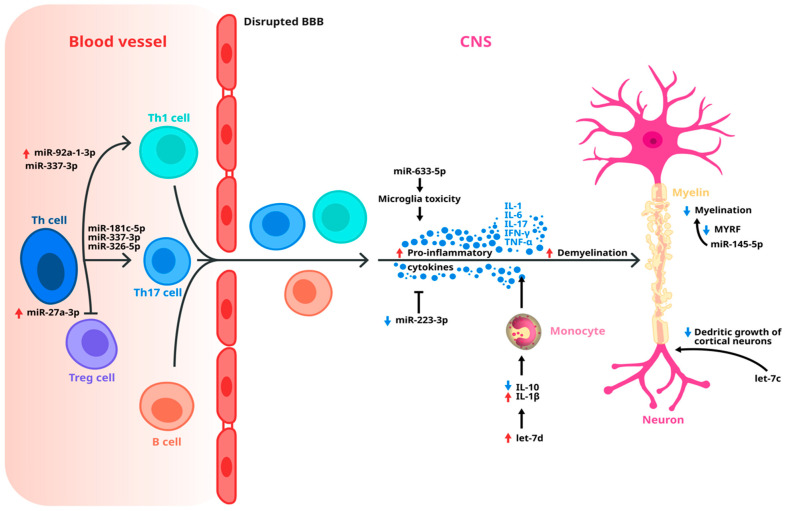
Circulating miRNAs involved in MS pathogenesis, which can indicate the transition from RRMS to SPMS (miR-92a-1-3p, miR-337-3p, miR-181c-5p, miR-326-5p, miR-27a-3p, miR-633-5p, miR-223-3p, miR-145-5p, let-7d, let-7c). Dysregulation of the miRNAs are marked as follows: the red arrows represent up-regulation of a specific miRNA in MS compared with a healthy control, the blue arrows represent down-regulation of a miRNA in MS compared with a healthy control. All the processes represented in this figure in the end lead to the destruction of the myelin sheath and disease progression.

While previous studies have established the definite involvement of miRNAs in MS pathogenesis (Figure 2), and we have highlighted the most clinically relevant ones, recent research focuses on identifying those that remain significant over extended periods. These efforts aim to uncover stable biomarkers for disease progression, which could enhance early diagnosis, monitoring, and personalized treatment strategies. In 2023, Agostini et al. investigated whether miRNAs could serve as predictive biomarkers for identifying patients at risk of progressing to SPMS. They conducted a retrospective observational study involving 69 RRMS patients, 36 of whom later transitioned to SPMS. The serum miRNA profile included miR-34a-5p, miR-103a-3p, and miR-376a-3p. After a 10-year follow-up, only miR-34a-5p and miR-376a-3p showed a significant increase in patients who progressed to SPMS [88].

In a recent study, Al-Temaimi et al. investigated miR-146-5p, miR-484, miR-343-3p, and miR-24-3p as potential biomarkers for the transition to SPMS. They concluded that the down-regulation of miR-24-3p could serve as a diagnostic marker for MS, while miR-146-5p and miR-484 were specifically down-regulated in SPMS patients [89].

Disease progression is assessed based on symptom severity and brain atrophy. Gonzalez-Martinez et al. monitored 144 RRMS patients, conducting biannual clinical examinations, annual MRI scans, and serum sample analyses. The results indicated that at the 10-year follow-up, patients with benign MS exhibited lower levels of miR-25-3p, while those who progressed to SPMS had higher levels of miR-320b, which also showed a positive correlation with the brain parenchymal fraction [90].

In addition to MRI, neurodegeneration could also be assessed through exosomal miRNAs, offering a potential non-invasive biomarker for monitoring disease progression and neuronal damage. Mohammadinasr et al. identified that the anti-inflammatory miR-132-5p and the pro-inflammatory miR-320a-5p were significantly down-regulated in both CSF and serum-derived exosomes of RRMS patients compared to HCs. Additionally, miR-150-5p and miR-342-3p, which have anti-inflammatory effects, as well as let-7g-5p, miR-18a-5p, miR-145-5p, and miR-374a-5p, which exhibit dual pro-inflammatory and anti-inflammatory actions, were significantly up-regulated in both CSF and serum-derived exosomes of RRMS patients compared to corresponding HCs [91].

## 6. Future Perspectives and Limitations of Exosomal miRNAs as a Biomarker for MS

Following these studies, there is strong evidence that miRNAs are seen by the researchers as an effective and reliable biomarker candidate used for monitoring the progression of MS (Figure 2). Exosomes are the most important miRNA carrier and could make the connection between the central compartment and the peripheral compartment that is so important in the MS physiopathology [92,93].

The sensitivity of exosomal miRNA diagnosis is supported by miRNAs’ stable expression and their resistance to the influence of external factors that could affect their expression, but also the development of high-throughput sequencing technology for determining the expression of miRNAs [94,95]. Some studies focused on the epigenetic markers that could be useful in the early diagnosis of MS and predict the prognosis of the disease. They isolated the miRNAs from animal models with EAE and determined that up-regulation of miRNAs is associated with antioxidant, anti-excitotoxicity, and pro-neuronal growth effects and a down-regulation of miRNAs associated with apoptosis [96,97].

However, even though the studies confirm the important role of miRNAs, the research is still in its early stages, especially when we talk about profiling the transition between RRMS and SPMS [98,99]. Isolation of miRNAs ha significant limitations, given the different methods of analysis (microarray, qRT-PCR, and NGS) and the variability of the biological source [100]. Exploring miRNA regulatory genes and target genes, moreover, and understanding their mechanism of action in MS pathogenesis are still challenges [101,102]. The complex network between miRNAs and the target transcript is a true test for researchers in their search to connect them with various disease entities [103,104].

Discovering new MS biomarkers should improve the management of this disease, with miRNAs having great potential for the research of new therapeutic targets [105,106]. Furthermore, the dysregulation of miRNAs in the exosomes, plasma, serum, and CSF of MS patients is an important step in finding biomarkers of disease prognosis and discrimination between clinical subtypes, thereby helping clinicians with therapeutic decisions or monitoring the therapeutic effects [107,108].

Although all these studies make us understand the great potential of miRNAs in the MS disease’s course and could discriminate between RRMS and SPMS, there is a need to confirm their effectiveness throughout clinical trials [109,110].

## 7. Conclusions

This review concentrated on the important implications of miRNAs and the role that they have in the physiopathology of MS. The complex pathogenesis of MS consists of genetic, epigenetic, exogenous, and environmental factors. Establishing a fast diagnosis of MS and monitoring the disease progression will be easier for clinicians with a simple, specific, and sensitive method of diagnosis. The analyses performed indicate that miRNAs could comprise a specific method to determine the disease’s condition stage. These findings highlight the potential clinical utility of miR-92a-1-3p, miR-337-3p, miR-181c-5p, miR-326-5p, miR-27a-3p, miR-633-5p, miR-223-3p, miR-145-5p, let-7d, and let-7c as valuable biomarkers for identifying and monitoring the progression from RRMS to SPMS. Furthermore, having a tool that could ease the diagnosis of the transition from RRMS to SPMS early is a considerable asset. When the disease progresses, a change in the therapeutic protocol is mandatory, offering a chance of a slower development of disease using a high-efficacy drug. New technology will offer a better understanding of the mechanisms of this complex disease and lead to the development of new drugs and treatment protocols that can target miRNAs and thus provide the patients with more chances for early diagnosis and treatment.

## Data Availability

Not applicable.
